# Supplementary material for: Synthesis, Physicochemical, Labeling and In Vivo Characterization of 44Sc-Labeled DO3AM-NI as a Hypoxia-Sensitive PET Probe
Source: Pharmaceuticals (Basel). 2022 May 26;15(6):666. doi: 10.3390/ph15060666 (PMC9228955; doi:10.3390/ph15060666)
Supplement: Supplementary file 1 [file pharmaceuticals-15-00666-s001.zip › pharmaceuticals-1706884-supplementary.pdf]

Supplementary Materials for

Synthesis, physicochemical, labeling and in vivo characterization of ^{44}Sc-labeled DO3AM-NI as a hypoxia sensitive PET probe

Dániel Szücs[1, 2, 3], Tibor Csupász[2,3], Judit P. Szabó[1, 4], Adrienn Kis[1], Barbara Gyuricza[1, 3], Viktória Arató[1, 5], Viktória Forgács[1, 3], Adrienn Vágner[6], Gábor Nagy[6], Ildikó Garai[1, 6], Dezső Szikra[1, 6], Imre Tóth[2], György Trencsényi[1, 6], Gyula Tircsó[2] and Anikó Fekete[1, *]

[1] Division of Nuclear Medicine and Translational Imaging, Department of Medical Imaging, Faculty of Medicine, University of Debrecen, Nagyerdei krt. 98., H-4032 Debrecen, Hungary; szucs.daniel@science.unideb.hu (D.S.); szabo.judit@med.unideb.hu (J.P.S); kis.adrienn@med.unideb.hu (A.K.); gyuricza.barbara@med.unideb.hu (B.G.); arato.viktoria@med.unideb.hu (V.A.); forgacs.viktoria@med.unideb.hu (V.F.); garai.ildiko@med.unideb.hu (I.G.); szikra.dezso@med.unideb.hu (D.S.); trencsenyi.gyorgy@med.unideb.hu (G.T.); fekete.aniko@science.unideb.hu (A.F.).

[2] Department of Physical Chemistry, Faculty of Science and Technology, University of Debrecen, Egyetem tér 1, H-4032 Debrecen, Hungary; csupasz.tibor@science.unideb.hu (T.C.); imre.toth@science.unideb.hu (I.T.); gyula.tircso@science.unideb.hu (G.T.).

[3] Doctoral School of Chemistry, Faculty of Science and Technology, University of Debrecen, Egyetem tér 1., H-4032 Debrecen, Hungary;

[4] Doctoral School of Clinical Medicine, Faculty of Medicine, University of Debrecen, Nagyerdei krt. 98., H-4032 Debrecen, Hungary;

[5] Doctoral School of Pharmaceutical Sciences, Faculty of Pharmacy, University of Debrecen, Nagyerdei krt. 98., H-4032 Debrecen, Hungary;

[6] Scanomed Ltd., Nagyerdei krt. 98., H-4032 Debrecen, Hungary; vagner.adrienn@scanomed.hu (A.V.); nagy.gabor@scanomed.hu (G.N.).

* Correspondence: fekete.aniko@science.unideb.hu (A.F.); Tel.:+36-52-255-510 (ext. 54470)

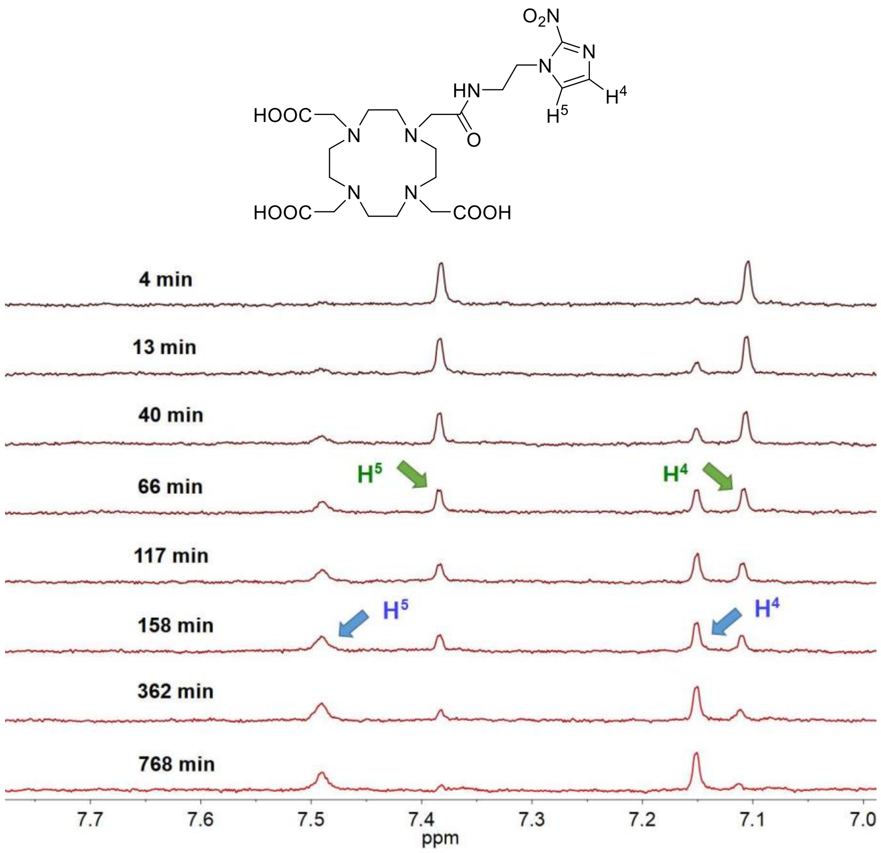

Figure S1. Aromatic region of the ^{1}H-NMR spectra of the samples used to probe the formation rate of Sc(DO3AM-NI) at pH = 1.52 (c_L=3.8 mM; $c_{Sc^{3+}}$=4.9 mM; I=0.15 M; 25 °C). Green arrows show the ^{1}H-NMR peaks of the ligand and blue arrows show the ^{1}H-NMR peaks of the scandium complex.

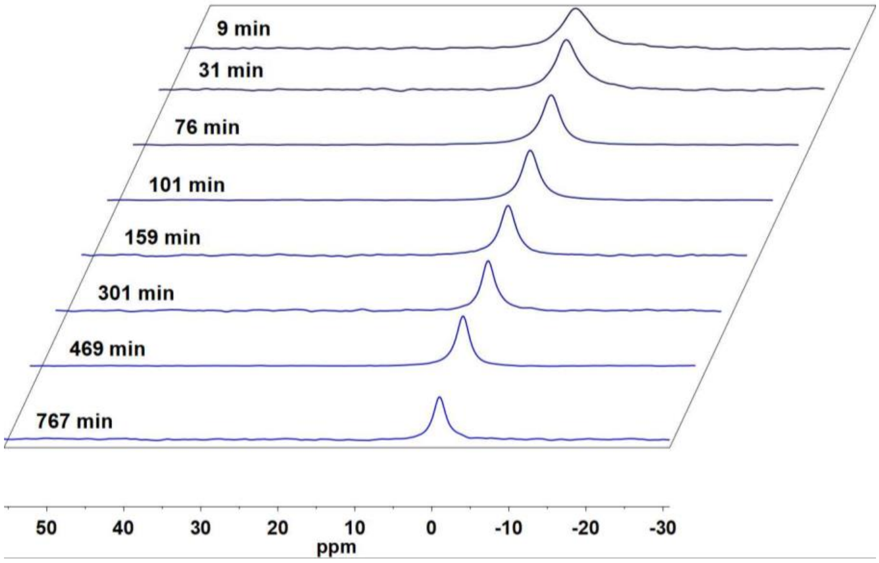

Figure S2. The ^{45}Sc-NMR spectra obtained as a function of time for the Sc(DO3AM-NI) complex formation at pH = 1.52 (c_L=3.8 mM; $c_{Sc^{3+}}$=4.9 mM; I=0.15 M; 25 °C) (δ=+4.8 − -0.7 ppm). These spectra show just the free $Sc^{3+}_{(aq)}$ peaks, the signal of the scandium complex is out of this chemical shift range.

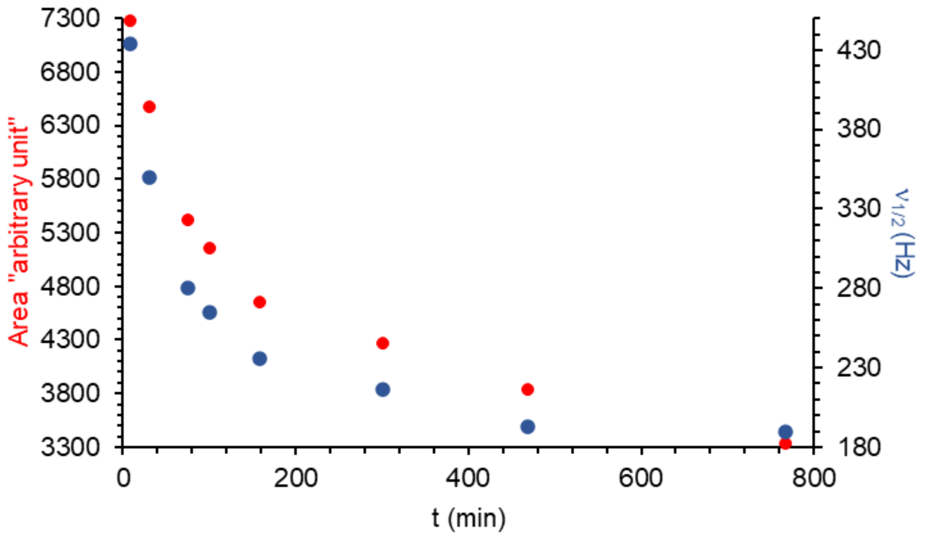

Figure S3. Peak area (●) and width (●) of the ^{45}Sc-NMR signal corresponding to the free Sc^{3+} as a function of time for the formation of Sc(DO3AM-NI) at pH = 1.52 (c_L=3.8 mM; $c_{Sc^{3+}}$=4.9 mM; I=0.15 M; 25 °C).

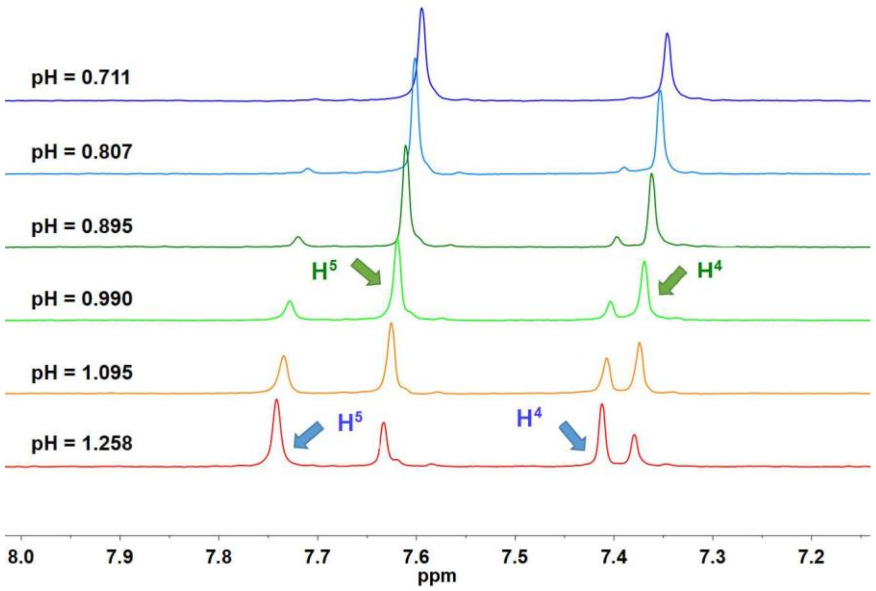

Figure S4. Aromatic region of the ^{1}H-NMR spectra of the samples used for the determination of the Sc(DO3AM-NI) complex (c_L=$c_{Sc^{3+}}$=3.2 mM; I=0.15 M; 37 °C). Green arrows show the ^{1}H-NMR peaks of the ligand and blue arrows show the ^{1}H-NMR peaks of the scandium complex.

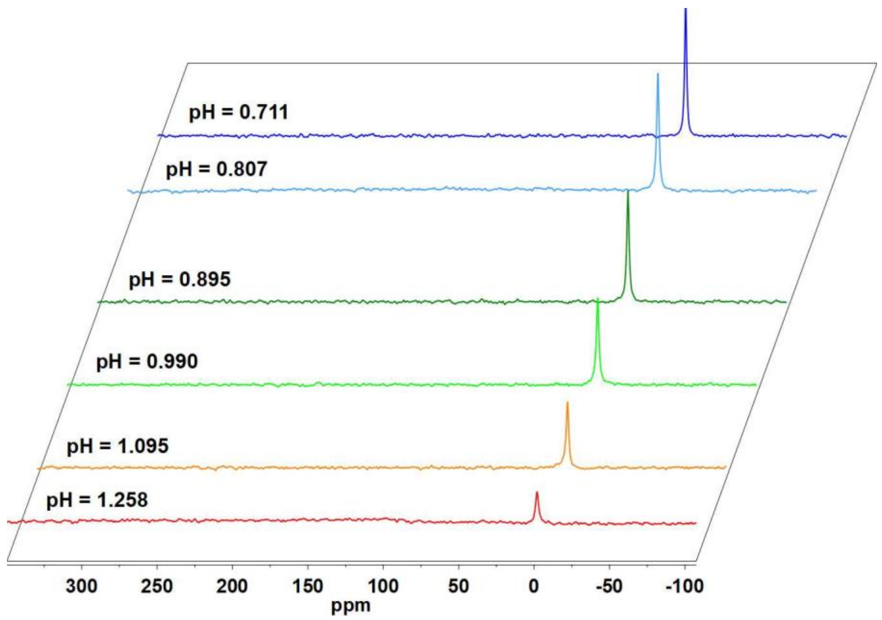

Figure S5. ^{45}Sc-NMR spectra of the samples used for the determination of the Sc(DO3AM-NI) complex (c_L=$c_{Sc^{3+}}$=3.2 mM; I=0.15 M; 37 °C).

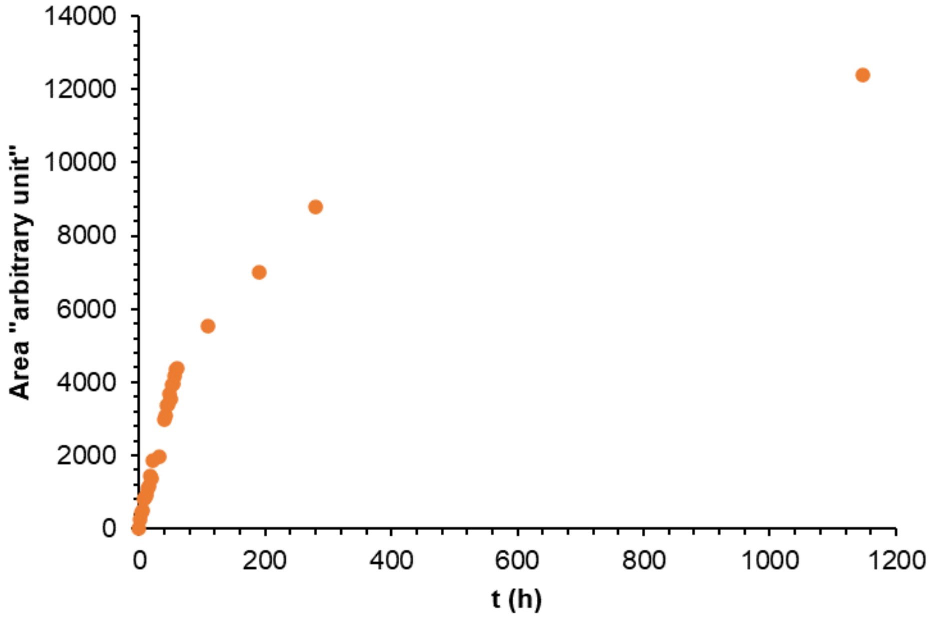

Figure S6. Peak area corresponding to the free Sc(III) as a function of time for the dissociation of Sc(DO3AM-NI) in 1.0 M HCl ($c_{complex}$=7.30 mM; I=1.03 M; 25 °C).

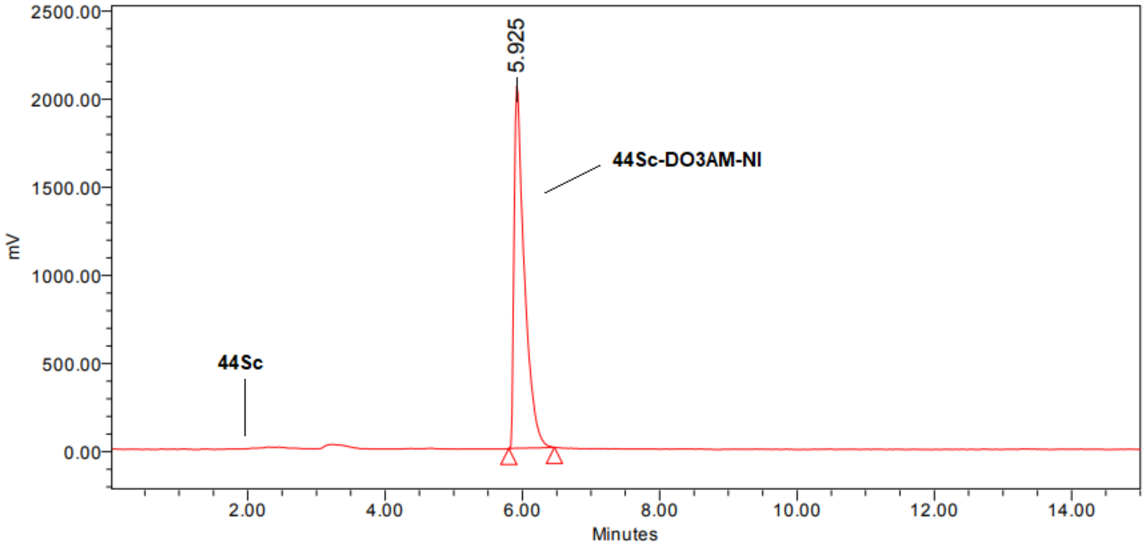

Figure S7. Radiochromatogram of purified [^{44}Sc]Sc(DO3AM-NI). Radio-HPLC was performed using a Luna C18 3 µm (150 x 4.6 mm) column, solvent A: oxalic acid (0.01 M pH=3); solvent B: acetonitrile.

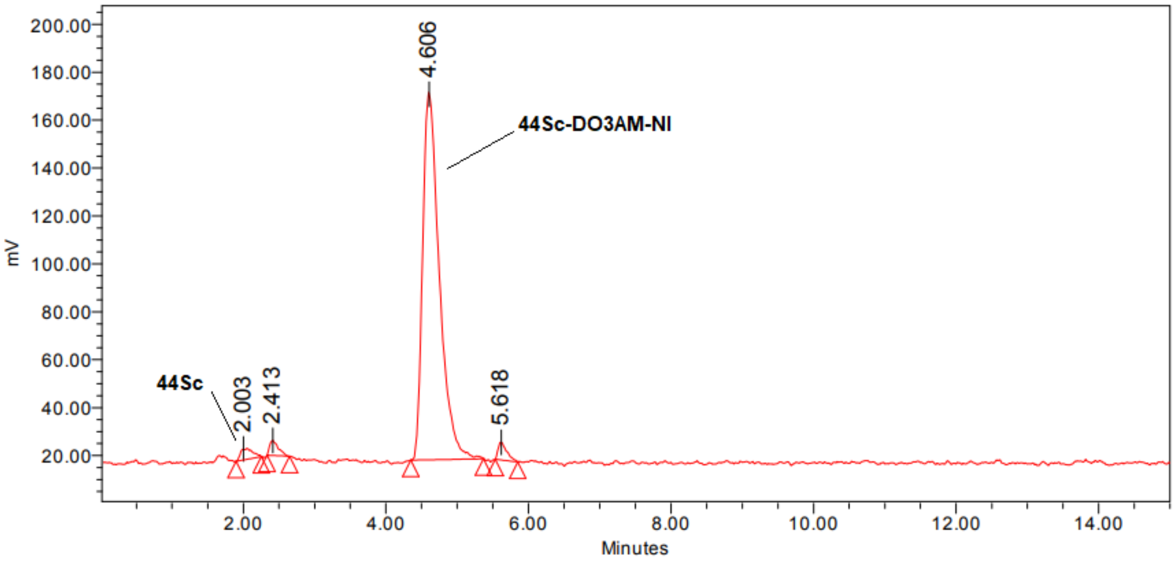

Figure S8. Radiochromatogram of the serum stability test of [^{44}Sc]Sc(DO3AM-NI) at 240 min. Radio-HPLC was performed using a Kinetex C18 2.6 µm (100 x 4.6 mm) column, solvent A: oxalic acid (0.01 M pH=3); solvent B: acetonitrile.
